# Case Report: True Motor Recovery of Upper Limb Beyond 5 Years Post-stroke

**DOI:** 10.3389/fneur.2022.804528

**Published:** 2022-02-17

**Authors:** Carine Ciceron, Dominique Sappey-Marinier, Paola Riffo, Soline Bellaiche, Gabriel Kocevar, Salem Hannoun, Claudio Stamile, Jérôme Redoute, Francois Cotton, Patrice Revol, Nathalie Andre-Obadia, Jacques Luaute, Gilles Rode

**Affiliations:** ^1^Service de Médecine Physique et Réadaptation, Plateforme Mouvement et Handicap, Hôpital Henry Gabrielle, Hospices Civils de Lyon, Pierre-Bénite, France; ^2^CRNL (Lyon Neuroscience Research Center, Trajectoires Team), INSERM U1028 & CNRS UMR 5292, Université Claude Bernard-Lyon 1, Bron, France; ^3^CREATIS, CNRS UMR 5220 & INSERM U1294, Université Claude Bernard-Lyon1, INSA de Lyon, Université de Lyon, Villeurbanne, France; ^4^CERMEP-Imagerie du Vivant, Université de Lyon, Bron, France; ^5^Medical Imaging Sciences Program, Division of Health Professions, Faculty of Health Sciences, American University of Beirut, Beirut, Lebanon; ^6^Service de Radiologie, Center Hospitalier Lyon-Sud, Hospices Civils de Lyon, Pierre-Bénite, France; ^7^Service de Neurologie Fonctionnelle et Epileptologie, Hôpital Pierre Wertheimer, Hospices Civils de Lyon, Bron, France; ^8^CRNL (Lyon Neuroscience Research Center, NeuroPain Team), INSERM U1028 & CNRS UMR 5292, University Claude Bernard-Lyon 1, Bron, France

**Keywords:** stroke, motor, recovery, upper-limb, dexterity, motion analysis, corticospinal tract, fMRI

## Abstract

Most of motor recovery usually occurs within the first 3 months after stroke. Herein is reported a remarkable late recovery of the right upper-limb motor function after a left middle cerebral artery stroke. This recovery happened progressively, from two to 12 years post-stroke onset, and along a proximo-distal gradient, including dissociated finger movements after 5 years. Standardized clinical assessment and quantified analysis of the reach-to-grasp movement were repeated over time to characterize the recovery. Twelve years after stroke onset, diffusion tensor imaging (DTI), functional magnetic resonance imaging (fMRI), and transcranial magnetic stimulation (TMS) analyses of the corticospinal tracts were carried out to investigate the plasticity mechanisms and efferent pathways underlying motor control of the paretic hand. Clinical evaluations and quantified movement analysis argue for a true neurological recovery rather than a compensation mechanism. DTI showed a significant decrease of fractional anisotropy, associated with a severe atrophy, only in the upper part of the left corticospinal tract (CST), suggesting an alteration of the CST at the level of the infarction that is not propagated downstream. The finger opposition movement of the right paretic hand was associated with fMRI activations of a broad network including predominantly the contralateral sensorimotor areas. Motor evoked potentials were normal and the selective stimulation of the right hemisphere did not elicit any response of the ipsilateral upper limb. These findings support the idea that the motor control of the paretic hand is mediated mainly by the contralateral sensorimotor cortex and the corresponding CST, but also by a plasticity of motor-related areas in both hemispheres. To our knowledge, this is the first report of a high quality upper-limb recovery occurring more than 2 years after stroke with a genuine insight of brain plasticity mechanisms.

## Introduction

Motor recovery usually occurs within the first 3 months after stroke and is more limited for upper limbs than lower limbs (1–6). Similarly, recent modeling showed that the probability to recover upper limb motor function is extremely limited after the first 12 weeks post-stroke onset (7), which can be explained by the damage sustained by the contralateral corticospinal tract (CST) and the limited vicarious capacities of the motor system to compensate for this complex and lateralized function, especially for the individual finger movement and manual dexterity (8). A patient with a complete motor deficit of the right upper limb after stroke was followed for 12 years. He presented a remarkable late recovery of the upper-limb motility, in terms of strength, individual finger movement, and manual dexterity, 5 years after stroke onset.

The aim of the present study was to assess objectively the upper-limb recovery of this patient over a long-time period and to explore the potential mechanisms underlying this unusually delayed recovery. For this purpose, 3D kinematic analysis were carried out as well as a neuro-anatomo-functional study of the CST, using diffusion tensor imaging (DTI), functional magnetic resonance imaging (fMRI), and transcranial magnetic stimulation (TMS) techniques.

## Case Description

The patient was a 53-year-old male, right-handed according to the Edinburgh laterality questionnaire. He underwent a stroke of cardio-embolic origin in the territory of the left superficial middle cerebral artery. He had an initial complete right hemiplegia and a severe mixed aphasia. He experienced intravascular thrombolysis 6 h after the onset of symptoms. His affected inferior limb recovered quite fast, and he was able to walk without limitation 3 months after the stroke, while he was unable to initiate movement in the right upper limb. He never had somato-sensory impairment nor spasticity. After 3 months of inpatient rehabilitation, he was discharged home and kept up with a standard physiotherapy of 30 min, 3 times a week. A home self-training program was monitored by the physiotherapist, including finger tapping exercises as soon as it was possible. The aphasia progressed, and he recovered oral comprehension, but a moderate expressive aphasia persisted. In the affected upper limb, the recovery was delayed and happened very gradually, along a proximal-distal gradient. Slight proximal movements, at the shoulder and elbow, re-appeared 6 months after the stroke onset. Slight global movements of the fingers were observed in the second year. A thumb-index finger grip was possible 4 years after the stroke, and gross prehension the following year. Six years after stroke onset, the movements were dissociated on the whole upper limb comprising the fingers. Nine years after the stroke, all kinds of prehension, gross, and fine, were functional. A brain MRI, performed 10 years after stroke onset, showed a single large infarction in the left superficial middle cerebral artery territory, sparing the cranial part of the precentral gyrus.

### Patient Perspective

The patient was well-informed about the prognosis of his stroke. He spontaneously reported at a follow-up consultation that he was able to move his fingers, conscious that it was unexpected at such a delay. When proposed to participate in a clinical study to characterize and better understand this recovery, he showed great interest. He has always been very active and involved in his rehabilitation. He resumed driving, tinkers and gardens regularly using both hands.

## Materials and Methods

Grip strength, manual dexterity, and function of the upper limb were assessed 3 months, and 5, 9, and 12 years after stroke using standardized tests. Grip strength was measured using a Jamar dynamometer (9). The strength considered was the mean of 6 trials. Manual dexterity was measured using Box and Blocks test (10) and Purdue Pegboard right hand subtest (11). The results are expressed as a percentage of the normal value for the corresponding age group. Motor function was measured using Fugl-Meyer Upper Extremity Scale (FMA-UE) (12).

### Kinematic Analysis of Reach-to-Grasp Movement

Repeated kinematic analyses of reach-to-grasp movement were performed at 5, 9, and 12 years after stroke onset. Results were compared to those of 6 right-handed control subjects (mean ± standard deviation (SD) age = 58.2 ± 5.5 years). Each participant sat facing a table. In rest position, the right hand laid on the table close to the trunk, in mid-pronation, thumb, and index finger in contact on the median line. A glass, 60 mm in diameter, was placed at 40 cm from the rest position, 20° to the right side of the median line.

Each participant was asked to take the glass with the right hand as naturally as possible and to lift it slightly. An alert-signal (red diode) flashed for 2 s to indicate the beginning of each trial. Then, a go-signal (green arrow) lit up, triggering the data acquisition. The beginning and end of each movement was determined by sensors placed under the rest position and under the glass. The glass was presented 11 times to each participant. We used a 3D motion capture system (Vicon 370®, Oxford, UK) with 5 infrared cameras to record the movement of 3 retro-reflective passive markers, placed on the thumb, index, and the internal radial styloid, at a frequency of 50 Hz. After recording and 3D reconstruction, the position of each marker was filtered with a Butterworth low-band pass filter, with a cut-off frequency of 6 Hz. Then, from the spatial position of the markers, movement parameters were computed using a homemade Handimain software. Relevant parameters related to the reach [movement time (MT), velocity peak (VP), time to velocity peak (TVP)], and grasp [maximal grip aperture (MGA) and time to maximal grip aperture (TMGA)] phases (13) were studied.

The Movement Time (MT) is the time between the initiation of the movement and the closure of the grip on the glass. The Velocity Peak (VP) is the maximal value of the wrist marker velocity during the movement. The Maximal Grip Aperture (MGA) measures the maximal distance between the thumb and index fingers during the grasp phase. These parameters were determined in a semi-automatic procedure with trial-by-trial validation by one expert experimenter. The trials for which the values were more or less than 2 standard deviations were removed.

### MRI and Transcranial Magnetic Stimulation (TMS)

Twelve years after stroke onset, the patient was examined by MRI and TMS.

Conventional anatomic MRI, DTI and fMRI were performed at the MRI department of CERMEP-Imagerie du vivant (Lyon, France) on a 1.5T Siemens Sonata MRI system (Siemens Medical Solutions, Erlangen, Germany). Ten healthy control subjects [mean ± SD (range) age = 47.7 ± 11.8 (30–66) years] were included in the fMRI study. All control subjects were right-handed, had a normal or corrected-to-normal vision, and had no history of neurological nor psychiatric disorders. The study was approved by the local ethics committee (CPP Sud-Est IV) and all participants gave their written informed consent.

Conventional 3D T1-weighted (T1w) images [repetition time (TR) = 2,120 ms, echo time (TE) = 3.9 s] of the brain were acquired using the following parameters: voxel size = 1 × 1 × 1 mm3, field of view (FOV) = 320 × 224 mm^2^, 384 axial slices.

DTI was performed to evaluate the integrity of corticospinal tracts (CST) using a 2D spin-echo echo-planar imaging diffusion sequence repeated twice (TR = 6,500 ms; TE = 86 ms; 24 diffusion-gradient orientations with b = 1,000 s.mm^−2^, 56 axial slices, FOV = 240 × 240 mm, voxel size = 2.5 × 2.5 × 2.5 mm3). The fractional anisotropy (FA) asymmetry index (FA-AI) was calculated from the left and right posterior limbs of the internal capsule (PLIC) and CST.

fMRI was performed using a finger opposition task of the thumb and the 4 other fingers. Statistical activation maps were created, first to contrast movement and rest in the patient as well as in each control subject, then to contrast movement and rest in the control group.

TMS was used to test the functional integrity of the ipsi-lesional corticomotor pathway following a methodology consistent with the International Federation of Clinical Neurophysiology guidelines (14).

The anatomic MRI, DTI, fMRI, and TMS methods are detailed in Appendix A (Supplementary Material online).

## Results

Grip strength, manual dexterity, and function of the paretic upper limb improved between the 5th and the 12th year post-stroke. Grip strength, manual dexterity and proximal motility of the paretic upper limb remained lower than normal at the 12th year, whereas distal motility reached the maximum score (Table 1). FMA-UE showed that proximal motility improved between 3 months and 5 years and then remained stable, whereas hand motility improved until the 12th year, indicating a proximo-distal gradient in recovery.

**Table 1:** Standardized clinical evaluation of the paretic hand of the patient, at 3 months, and 5, 9, and 12 years post-stroke.

**Delay from stroke onset**	**3 mths**	**5 yrs**	**9 yrs**	**12 yrs**
**Fugl-Meyer Upper Extremity Scale (/66)**	0	46	54	56
Proximal motility (/36)	0	26	26	26
Wrist motility (/10)	0	9	10	10
Hand motility/prehension (/14)	0	8	12	14
Coordination and speed (/6)	0	3	6	6
**Grip strength (kg)**	NM	NM	25 (40.8; 61%)	33 (41.4; 80%)
**Manual dexterity**				
Box and blocks test	NM	26 (75.2; 35%)	32 (71.3; 45%)	42 (68.4; 61%)
Purdue pegboard right hand subtest	NM	NM	5.5 (13.6; 42%)	6.5 (13.6; 49%)

*mths, months; yrs, years; NM, non-measurable. For Fugl-Meyer Upper Extremity Scale, brackets indicate the maximum score. For grip strengh and manual dexterity, brackets indicate the normal value for the age group and the percentage of the normal value*.

### Kinematic Analysis of Reach-to-Grasp Movement

**Figure 1:**
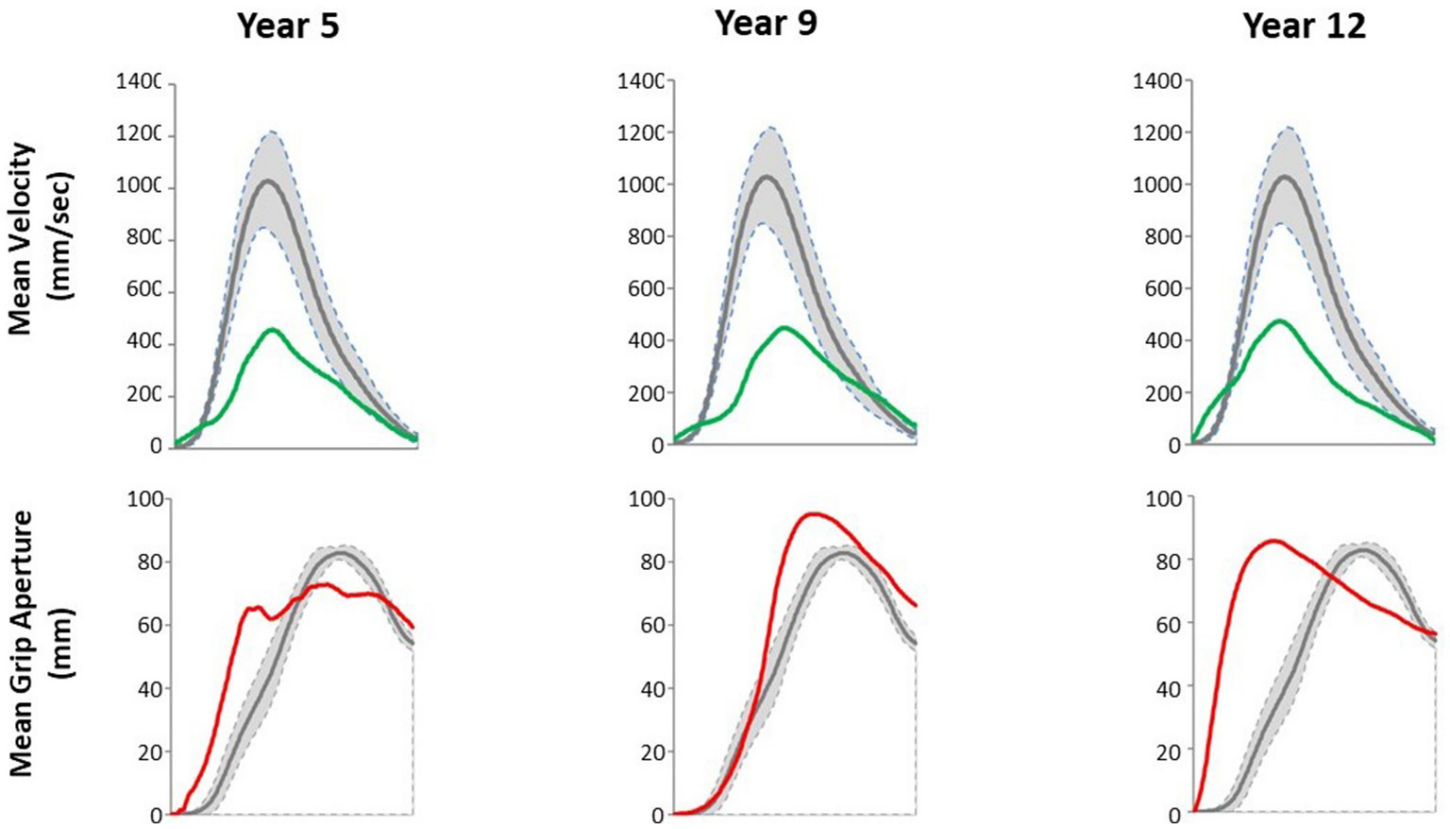
Mean velocity and mean grip aperture profiles relative to a standardized prehensile movement time for a glass located on the right side for the patient (green and red) and in a group of six healthy controls (gray). In the control group mean ± SD is plotted.

Data collected at 5, 9, and 12 years post-stroke onset are presented in Table 2, Figure 1.

**Table 2:** Kinematic parameters of the reach-to-grasp movement for the paretic upper limb of the patient and for the right upper limb of 6 control subjects.

	**Patient**			**Controls**
	**Y5**	**Y9**	**Y12**	
**Mean** ***(SD)***				
**Transport phase**				
MT (msec)	2,805.7 *(724.2)*	1,572.5 *(149.2)*	1,740.9 *(156.5)*	1,004.7 *(84.6)*
VP (mm/sec)	513.0 *(39.5)*	474.9 *(29.0)*	481.4 *(25.3)*	1,098.4 *(96.0)*
TVP/MT (%)	15.8 *(7.4)*	35.4 *(3.2)*	32.0 *(2.7)*	39.5 *(2.6)*
**Grasp phase**				
MGA (mm)	108.8 *(7.5)*	136.3 *(5.0)*	123.9 *(7.4)*	118.6 *(4.7)*
TMGA/MT (%)	51.6 *(22.8)*	57.8 *(6.0)*	42.5 *(3.9)*	69.7 *(5.6)*

*Y5, Y9, Y12 correspond to 5, 9, and 12 years after stroke onset. MT, movement time; VP, velocity peak; TVP, time to velocity peak; MGA, maximum grip aperture; TMGA, time to maximum grip aperture. The italic values correspond to standard deviations of each parameter*.

Regarding the reach component, mean MT of the patient was 279% of the control value at the 5th year assessment. It decreased by 44% between the 5th and the 9th year and then increased by 11% between the 9th and the 12th year. It was 157% of the control value at the 9th year and 173% of the control value at the 12th year assessment. VP remained lower in the patient than in controls by 53–57% at all time points. Regarding the grasp component, mean MGA was 92% of the control value at the 5th year assessment. It increased by 26% between the 5th and the 9th year and then decreased again by 9% between the 9th and the 12th year. It is 104% of the control value at the 12th year. Moreover, the shape of the grip aperture curve of the patient became smoother and more similar to that of the control subjects from the 5th to the 12th year.

### DTI

**Figure 2:**
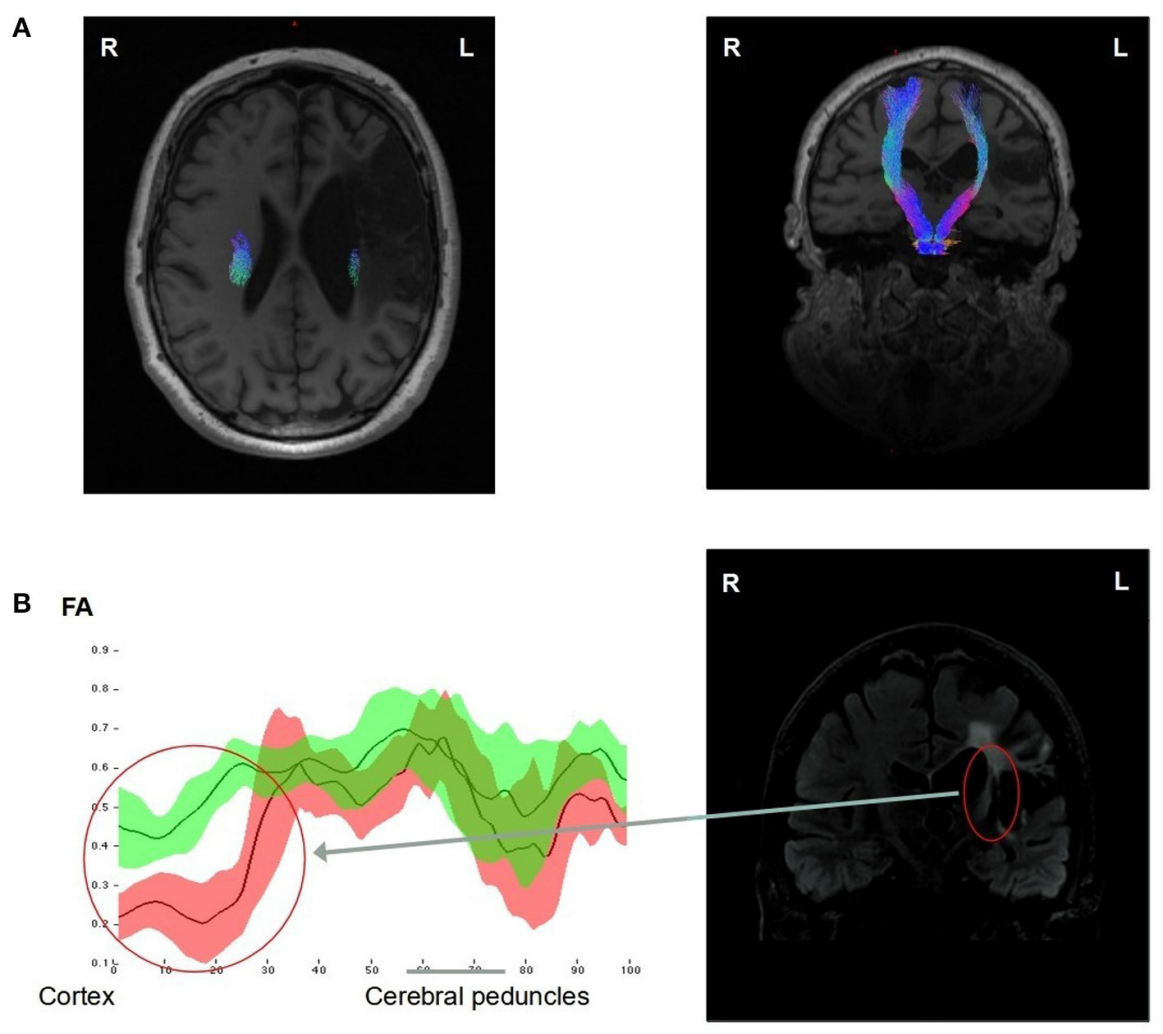
DTI analysis of the corticospinal tracts (CST) of the patient 12 years after stroke onset. The CSTs of both hemispheres were reconstructed and represented in the axial and coronal T1w MRIs showing a reduction of the fiber numbers in the left CST compared to the right, particularly in its upper part, going between the ischemic lesion and the lateral ventricle **(A)**. FA measures (mean ± 1 SD) along both CST profiles, going from the up cortex to the down cerebral peduncles regions, showed a significant decrease (>2 SD) in the left (red) CST compared to the right (green) CST, in the upper part of the left CST (Red circle) **(B)**.

As illustrated in Figure 2, the FA analysis, performed along the CST profile, differentiated two parts. In the upper part, where the CST goes between the lesion and the lateral ventricle, the FA was significantly lower in the left tract compared to the right. This argues for an alteration of structural integrity of the CST limited to the infarcted area. In the lower part, the FA was not significantly different between the right and left tracts, indicating that this alteration was not extended downstream of the infarction. The patient FA asymmetry index (FA-AI) measured at the level of the PLIC was equal to 0.423.

### fMRI

**Figure 3:**
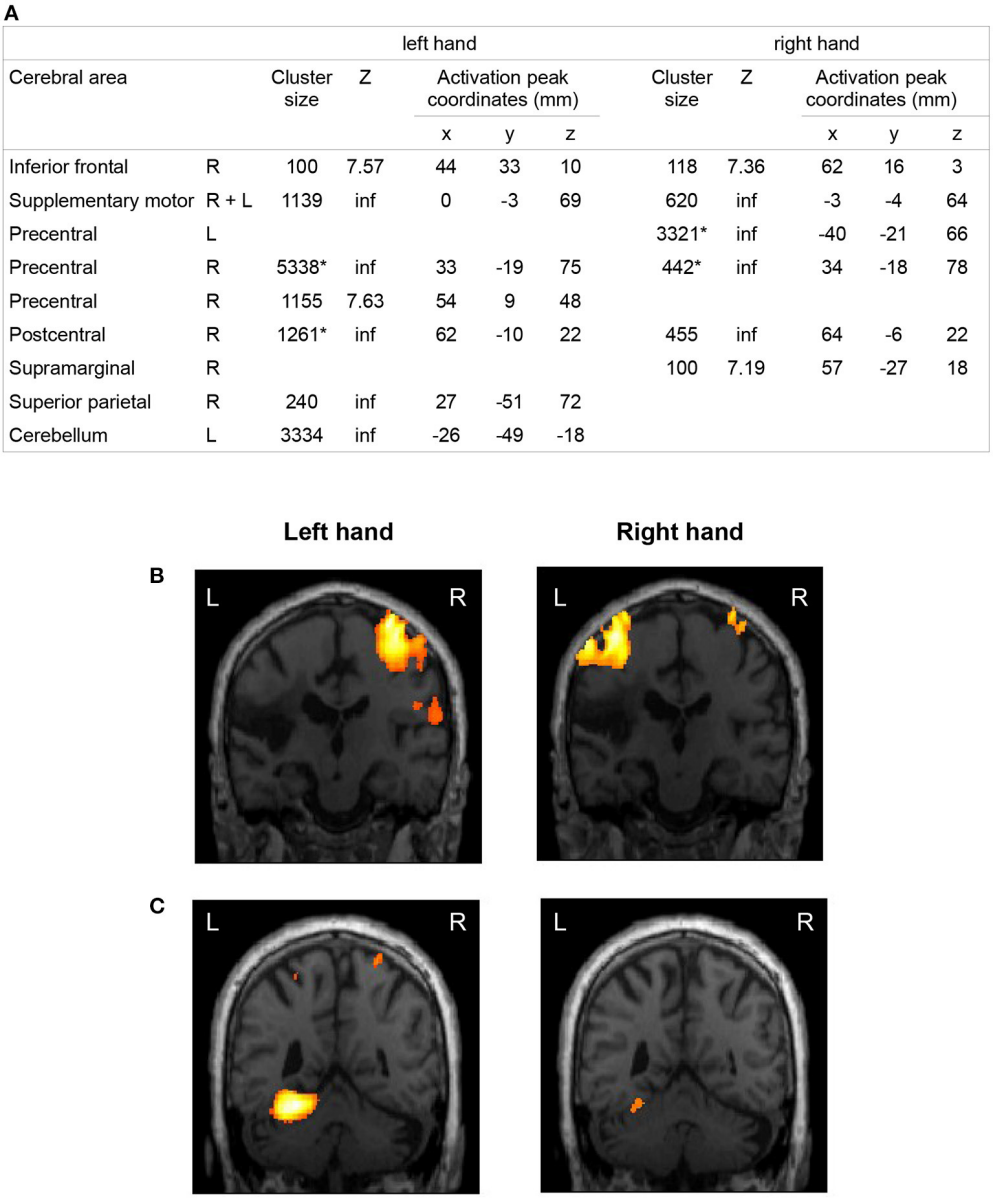
fMRI activations during a finger opposition movement in the patient 12 years after stroke. Brain areas activated by the movement of the left unaffected hand and the right affected hand of the patient compared to rest. The coordinates of the local maximum and the *Z* score were displayed for each significantly activated cluster of more than 100 voxels (*p* < 0.001, FWE corrected). The origin of the coordinates is at the anterior commissure in the Talairach space. R, right; L, left; inf, infinite. *Clusters including both pre- and post-central gyri **(A)**. Activation maps were represented in the coronal plane corresponding to the activation peak of the cluster (*p* < 0.001, FWE corrected at the voxel level) of the contralateral primary motor cortex **(B)** and the cerebellum **(C)** during the left and right hand exercise (L, left; R, right).

The patient performed the task correctly with both hands. While he performed finger movements with the right impaired hand, fingers from his left hand displayed slight involuntary movements during most of the run, consistent with mirror movements. Significant brain activations during the finger opposition task in the patient are reported in Figure 3.

The finger movement of both hands was associated with widespread activations in the contralateral sensorimotor areas. The finger movement of the right impaired hand additionally elicited a small activation of the ipsilateral sensorimotor areas. While the activation of the right motor area elicited by the left hand movement covered a large part of the precentral gyrus, the activation of the left motor area elicited by the right affected hand movement was limited to the upper part of the precentral gyrus, the lower part being infarcted. A wide activation was observed in the ipsilateral cerebellum during the finger movement of the left hand, but not during the finger movement of the right affected hand.

For comparison, brain activations during the finger opposition task observed in the control group are reported in Supplementary Table (Supplementary Material online).

### TMS

Motor evoked potentials were normal at both upper limbs, without any asymmetry regarding central conduction time and amplitude of responses to transcranial magnetic stimulation. When stimulating selectively one hemisphere with the butterfly coil under neuro-navigation, the cortical excitability threshold was estimated at 57% in the left hemisphere and 70% in the right hemisphere. With a stimulation at 120% of motor threshold, no response of the upper limb ipsilateral to the stimulation was elicited, neither at rest nor facilitated by voluntary contraction of the target muscle.

## Discussion

Although affected by a complete motor deficit of the right upper limb immediately after stroke, which is known to be a prognostic factor of poor recovery (15), our patient presented a remarkable recovery of upper-limb motility including strength, individual finger movement, and manual dexterity 5 years after stroke onset. This recovery took place much later than usually reported in the literature (8).

As recommended by Kwakkel et al. (16), standard clinical measures were associated with quantified movement analysis herein to better discriminate between neurological recovery and behavioral compensation. The evolution of FMA-UE between the 3rd month and the 12th year showed a motor recovery according to a proximo-distal gradient corresponding to the most frequent recovery profile after a supratentorial stroke (12, 17). Grip strength was measurable only after 5 years, which argues against a regression of non-use (18) or a motor neglect (19). Likewise, fine prehension movements required for Purdue Pegboard test became possible only after 5 years.

Kinematic analysis findings showed that the movement parameters of the paretic hand improved over time. MT representative of the reach phase decreased between the 5th and the 9th year, consistently with the FMA-UE that showed an improvement of proximal motility during the first 5 years after stroke. MGA, representative of the grasp phase, was lower than in controls at the 5th year, as observed in patients with a severe distal impairment. It became larger than in controls at the 9th year, as observed in patients with mild to moderate distal impairment (20). It finally decreased to approach the control value at the 12th year, arguing for an improvement of distal motility over the 12 year period (13). The improvement in motor scores and movement characteristics is more in favor of a true motor recovery than a compensation process taking advantage of the preserved proximal motility (21). The recovery of dissociated finger movements could be explained by the re-establishment and/or reorganization of anatomo-functional brain areas involved in motor control of the right hand and fingers and a left functional CST (8, 22).

DTI and TMS findings supported this hypothesis. As shown by the FA measurement, an alteration of the integrity of the left CST was found in the upper portion of the tractus. DTI tractography showed that the left CST was thinner than the right CST, but some of the fibers were preserved all along the left CST, from the primary motor cortex to the cerebral peduncle. The left CST displayed signs of injury but presumably regained some functionality, as demonstrated by the motor evoked potentials. This finding is in line with the literature showing that recovery of selective finger movements is dependent on CST integrity (23, 24).

The fMRI study showed that the network activated during the finger movement of the right affected hand comprised motor and non-motor brain regions of both hemispheres. Among these regions, a large activation was located in contralateral sensorimotor areas, which is known to be associated with a good motor recovery (25). This network is broader than the network activated by the same movement performed with the non-affected hand. This is in line with previous imaging studies and suggests the recruitment of additional areas to compensate for the partial lesion of the motor cortex and the CST (26–31). The activation of sensori-motor regions of the ipsilateral hemisphere during the movement of the right affected hand could also be related to mirror movements of the left upper limb during the task (32, 33). The ipsilateral cerebellum was activated during the finger movement of the left unaffected hand in the patient, but not during the finger movement of the right affected hand. This observation can be interpreted as a persistent crossed cerebellar diaschisis (34). Due to the small number of control subjects compared to a single patient, the comparison between patient and controls has limited value and will not be discussed here.

In summary, these findings add to emerging evidence that, in some patients, motor recovery of the upper limb may not be restricted to the first 3 to 6 months after stroke onset. From a sample of 219 individuals with mild-to-moderate upper limb hemiparesis, an extension of this critical time window for recovery has already been demonstrated up to 18 months post-stroke (35). In the SALGOT study, a few patients showed improvement in FMA-UE or Action Research Arm Test between 3 and 12 months post-stroke (36). Bach-y-Rita had already reported a significant motor recovery over a 5-year period after a brainstem infarct in a 65 year old patient. Common points with our patient were an extensive home rehabilitation program, a strong motivation and a very active life (37). Sörös et al. described some recovery of the upper limb motricity 23 years after stroke in a young man, but they did not give information about fine motricity of the fingers (38). Stinear et al. (39) have reported that CST integrity is a predictor of functional potential in chronic stroke patients. Indeed, in patients with motor evoked responses to TMS in the affected upper limb, an intensive rehabilitation program can lead to meaningful gains 3 years after stroke. Although some cases of unusually late recovery have already been reported, it is the first time such a good quality recovery from a complete paralysis is described in this time frame and it is so precisely studied. This remarkable recovery could be explained by the combined restoration of nerve conduction in the affected CST (40) together with a cortical brain reorganization rather than the involvement of the opposite CST (41, 42). This might be explained by the removal of a central conduction block analogous to neuropraxia in the peripheral nervous system (43).

This remarkable late motor recovery of upper limb invites experts and physicians to temper their statements regarding the time course of recovery after stroke. It also highlights the interest of combining different techniques such as quantified movement analysis, structural and functional imaging, and electrophysiology for an extensive understanding of exceptional cases.

## Data Availability Statement

The original contributions presented in the study are included in the article/Supplementary Material, further inquiries can be directed to the corresponding authors.

## Ethics Statement

The studies involving human participants were reviewed and approved by CPP Sud-Est IV. The patients/participants provided their written informed consent to participate in this study.

## Author Contributions

CC: data analysis and writing. JL and GR: conception of the study, monitoring, proofreading, and technical advice. NA-O: TMS, data analysis and technical advice, and proofreading. PRe: quantified movement analysis, data analysis and technical advice, and proofreading. FC: technical advice about MRI. JR: technical advice about fMRI data analysis. GK, SH, and CS: DTI, data analysis and technical advice. SB: fMRI control subjects and data analysis. PRi: quantified movement analysis. DS-M: technical advice, monitoring, and proofreading. All authors contributed to the article and approved the submitted version.

## Conflict of Interest

The authors declare that the research was conducted in the absence of any commercial or financial relationships that could be construed as a potential conflict of interest.

## Publisher's Note

All claims expressed in this article are solely those of the authors and do not necessarily represent those of their affiliated organizations, or those of the publisher, the editors and the reviewers. Any product that may be evaluated in this article, or claim that may be made by its manufacturer, is not guaranteed or endorsed by the publisher.

## Abbreviations

CST: corticospinal tract
DTI: diffusion tensor imaging
fMRI: functional magnetic resonance imaging
FA: fractional anisotropy
FMA-UE: Fugl-Meyer Upper Extremity Scale
PMC: premotor cortex
PLIC: posterior limb of the internal capsule
ROI: region of interest
SMA: supplementary motor area
TMS: transcranial magnetic stimulation.
