# Supplementary material for: Case Report: True Motor Recovery of Upper Limb Beyond 5 Years Post-stroke
Source: Front Neurol. 2022 Feb 17;13:804528. doi: 10.3389/fneur.2022.804528 (PMC8891374; doi:10.3389/fneur.2022.804528)
Supplement: Supplementary file 1 [file Table_1.pdf]

Table 1: Brain areas activated during a finger opposition movement of the left and right hands in ten healthy control subjects compared to rest.

		Left hand					Right hand				
Cerebral area		Cluster size	Z	Activation peak coordinates (mm)			Cluster size	Z	Activation peak coordinates (mm)		
				x	y	z			x	y	z
Supplementary motor	R						107	4.72	6	2	64
Precentral	R	1986	5.6	33	-13	52					
	L						999	5.31	-32	-18	58
Frontoparietal operculum	R	105	5.25	54	-21	19					
	L						729	5.4	-51	-22	13
Cerebellum	R						656	5.75	12	-60	-12
	L	1191	5.36	-24	-49	-23					

The coordinates of the local maximum and the Z score are displayed for each significantly activated cluster of more than 100 voxels ($p < 0.05$, FWE corrected).The origin of the coordinates is at the anterior commissure in the Talairach space.
Abbreviations: R, right; L, left.
