# Supplementary material for: Case Report: True Motor Recovery of Upper Limb Beyond 5 Years Post-stroke
Source: Front Neurol. 2022 Feb 17;13:804528. doi: 10.3389/fneur.2022.804528 (PMC8891374; doi:10.3389/fneur.2022.804528)
Supplement: Supplementary file 2 [file Data_Sheet_1.pdf]

Appendix A: MRI and TMS methods

Twelve years after stroke onset, the patient was examined by MRI and TMS.
Conventional anatomic MRI, DTI and fMRI were performed at the MRI department of CERMEP-Imagerie du vivant (Lyon, France) on a 1.5T Siemens Sonata MRI system (Siemens Medical Solutions, Erlangen, Germany). Ten healthy control subjects (mean ± SD (range) age = 47.7 ± 11.8 (30-66) years) were included in the fMRI study. All control subjects were right-handed, had a normal or corrected-to-normal vision, and had no history of neurological nor psychiatric disorders. The study was approved by the local ethics committee (CPP Sud-Est IV) and all participants gave their written informed consent.

Conventional 3D T1-weighted (T1w) images (repetition time (TR) = 2120 ms, echo time (TE) = 3.9 s) of the brain were acquired using the following parameters: voxel size = 1 x 1 x 1 mm^3, field of view (FOV) = 320 x 224 mm^2, 384 axial slices.

DTI

DTI was performed to evaluate the integrity of corticospinal tracts (CST) using a 2D spin-echo echo-planar imaging diffusion sequence repeated twice (TR = 6500 ms; TE = 86 ms; 24 diffusion-gradient orientations with b = 1000 $s.mm^{-2}$, 56 axial slices, FOV = 240 x 240 mm, voxel size = 2.5 x 2.5 x 2.5 mm^3).
After movement correction, the fractional anisotropy (FA) map was calculated using the FDT module of FSL. FA is a unit-less measure of the directionality of water diffusion comprised between 0 and 1. It is 0 in a liquid compartment, where the diffusion is isotropic, and close to 1 in a dense white matter tract, where the diffusion is almost unidirectional. ROIs corresponding to the left and right posterior limbs of the internal capsule (PLIC) were delimited using the ICBM-81 DTI atlas. Each ROI was then eroded manually to remove any adjacent grey matter. The structural integrity of the PLIC was quantified by calculating a FA asymmetry index (FA-AI) from the mean FA values of the ipsilesional (FAipsi) and of the contralesional (FAcontra) PLIC, as described by Stinear et al. (1):

$$FA\text{-}AI = (FAcontra – FAipsi) / (FAcontra + FAipsi)$$

A probabilistic tractography of the left and right CST was carried out using the BEDPOST tool in FSL. Both CST were localised using the white matter atlas ICBM DTI-81 and co-registered to the FA map of the patient. Before proceeding to tractography, the fiber orientation distribution function was computed in each voxel using the constrained spherical deconvolution method (2). In order to define and analyse the white mater fiber-tracts related to the activated grey matter regions detected during the fMRI task (described below), the fMRI activation maps were co-registered to the FA map using NiftyReg (UCL Centre for Medical Image Computing, London, United Kingdom). The white matter fiber-tracking of CST were then generated from the ROIs corresponding to the activated areas of the primary sensorimotor cortices and supplementary motor areas using the probabilistic tractography module in Mrtrix (Brain Research Institute, Melbourne, Australia, http://www.brain.org.au/software/) (10 000 fibers per tract; minimum bending radius: 2 mm). Each fiber-track was divided in 100 slices of equal thickness. Mean and standard deviation of FA were calculated for each slice as represented in Figure 2.

fMRI

All participants were asked to perform an opposition movement of the thumb with the other four fingers successively. The pacing tone was presented every 3 seconds for the left hand of the patient and for both hands of control subjects, and every 5 seconds for the right hand of the patient. For each hand, a block design experiment was used. It was composed of seven 30s-blocks of movement alternated with seven 30s-blocks of rest.
T2*-weighted blood oxygenation level-dependent (BOLD) echo-planar imaging sequences were acquired (TR = 2500 ms, TE = 60 ms, FOV = 220 x 220 mm^2, slice thickness 5 mm, matrix size = 64 x 64; voxel size = 3.4 x 3.4 x 5 mm^3). The slices were acquired parallel to the anterior-posterior

commissure line.

fMRI data were analysed using Statistical Parametric Mapping (SPM 8, Wellcome Trust Centre for Neuroimaging, London, United Kingdom) implemented in Matlab7 (The MathWorks, Inc., Natick, Massachussetts). All images were realigned to the first image in order to correct for interscan movements. The observed translations were smaller than 3 mm, and rotations were smaller than 2 degrees. Images were normalised to the Montreal Neurological Institute (MNI) stereotactic atlas using non-linear transformation parameters obtained during the segmentation in different tissue classes of the anatomical T1 image. Images were then smoothed with an isotropic 8 mm full-width half maximum Gaussian kernel. For the patient, a mask of the lesion was manually drawn using the MRIcron® software (Mc Causland Center, Columbia, SC).
Statistical analysis was performed in two stages. First, a statistical activation map was created for each participant. The BOLD signal was convolved with a canonical haemodynamic response function and then used in a general linear model. This allowed to contrast all of the brain volumes acquired during the movement blocks with those acquired during the rest blocks (movement – rest). For the individual analysis in the patient, the statistical threshold ($p<0.001$) was corrected for multiple comparisons. Only clusters of more than 100 voxels were considered.
A second analysis was carried out in the control group, including all the contrasts gathered in every single subject in a 1-sample-t-test, in order to obtain statistical activation maps for the group (thresholded at $p<0.05$ and corrected for multiple comparisons). Only clusters of more than 100 voxels were considered.

TMS
A Mag-Pro X100 (MagVenture©) stimulator was used to deliver transcranial and paravertebral stimuli, through an MMC-140-II coil. Cortical stimulation was performed with the coil centred over the vertex. Spinal stimulation was performed with the coil centred over C7 to activate cervical roots. Peripheral motor conduction was also assessed by electrical stimulation of ulnar nerve at the wrist to record motor distal latency, F-wave latency and peripheral motor response maximal amplitude. Motor evoked potentials (MEPs) were recorded (Medelec Synergy) from the abductor digiti minimi muscles.
Central motor conduction time was measured using two methods: motor root stimulation and F-wave technique. The amplitude ratio was calculated between responses to cortical magnetic and to peripheral electrical stimulation.
Upper limbs resting motor threshold was determined at rest, with a figure of eight coil (cool-B65 butterfly shape coil MagVenture©) (3). The motor hot spot was localised under Neuronavigation (Visor2TM ANT neuro©).

References:

1. Stinear CM, Barber PA, Smale PR, Coxon JP, Fleming MK, Byblow WD. Functional potential in chronic stroke patients depends on corticospinal tract integrity. Brain J Neurol. jan 2007;130(Pt 1):170-80.

2. Tournier J-D, Calamante F, Connelly A. Robust determination of the fibre orientation distribution in diffusion MRI: non-negativity constrained super-resolved spherical deconvolution. NeuroImage. 1 may 2007;35(4):1459-72.

3. Rossini PM, Barker AT, Berardelli A, Caramia MD, Caruso G, Cracco RQ, et al. Non-invasive electrical and magnetic stimulation of the brain, spinal cord and roots: basic principles and procedures for routine clinical application. Report of an IFCN committee. Electroencephalogr Clin Neurophysiol. aug 1994;91(2):79-92.
